# Sigmoid and cecum colon volvulus: a case report

**DOI:** 10.1186/s13256-024-04622-z

**Published:** 2024-06-29

**Authors:** Dilawer Chofan Charo, Fares Medhat Mohammad, Mahmoud Issam Ghmera, Batoul Aksam Saker, Ayman Ali Ghosnah

**Affiliations:** 1 General Surgery Department, Ministry of Health, Latakia, Syria; 2 Medical Research Group of Egypt (MRGE), Negida Academy, Arlington, MA USA

**Keywords:** Colon volvulus, Sigmoid volvulus, Cecum volvulus, Double colon volvulus, Case report

## Abstract

**Introduction:**

Colon volvulus is the twisting of a segment of colon on its mesenteric axis, which can lead to the obstruction of the lumen and the blood supply. Colon volvulus is common in “volvulus belt” countries and can involve the sigmoid (60–70%) and cecum (25–40%).

**Case presentation:**

We report a case of a 47-year-old male, Alawites, who presented with bowel obstruction and dilated abdomen without any specific abdominal pain. Abdominal laparotomy showed both sigmoid and cecum volvulus with no signs of perforation or ischemia.

**Discussion and conclusion:**

One of the possible risk factors of sigmoid colon volvulus is the length of the rectum and sigmoid, while mobile cecum is considered as a possible reason for cecum volvulus. The management remains controversial and is specific for every case, depending mainly on the vitality of the colonic walls and the general condition of the patient.

## Introduction

Colon volvulus (CV) refers to the twisting of a segment of colon on its mesenteric axis. This twisting can lead to the obstruction of the lumen and the blood supply and may led to ischemia or perforation of the affected segment of colon [1]. CV is the third leading cause of large intestinal obstruction following colorectal cancers and complicated sigmoid diverticulitis. CV is more common in some countries than in others; these countries are called the “volvulus belt” and include the Middle East, India, Africa, South America, Russia, and Eastern Europe. These distribution of CV in these regions may be due a possible correlation with dietary, lifestyle, and genetic factors that contribute to the development of a redundant colonic segment prone to volvulus [2].

Volvulus can occur in any colonic segment that has sufficient redundancy to twist upon itself. It most commonly involves the sigmoid (60–70% of all cases), and the cecum is the second most common site, involved in 25–40% of all cases [3]. Other segments of the colon are rarely affected, but, when they are, the clinical presentation can be atypical and the diagnosis more challenging. A particularly rare and complex form of this condition is the double colon volvulus, where two separate segments of the colon twist simultaneously. This occurrence is exceedingly uncommon and poses a significant challenge in both diagnosis and management [4]. Understanding of the pathophysiology, epidemiology, and clinical implications of CV is crucial for healthcare professionals, especially in high-prevalence areas. This case report has been reported in line with the SCARE criteria [5].

## Case presentation

**Fig. 1:**
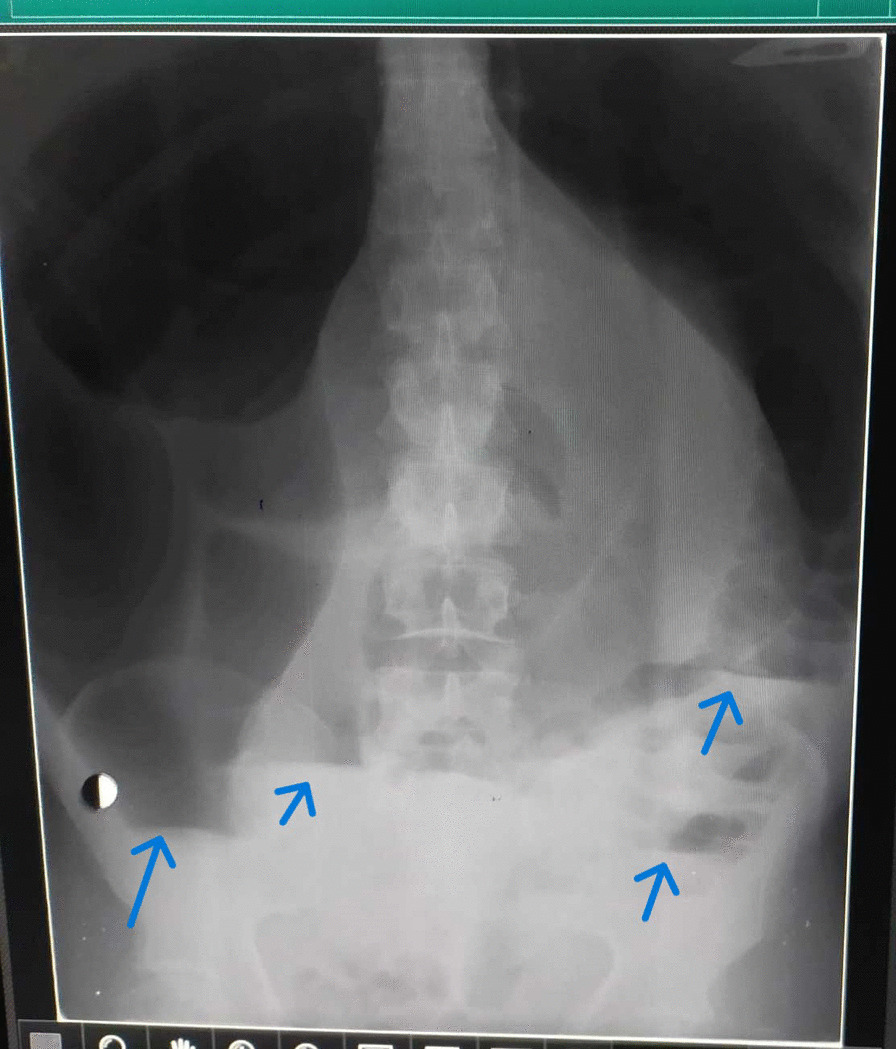
Abdominal X-ray showing extremely dilated colon and air–fluid levels (blue arrows)

**Fig. 2:**
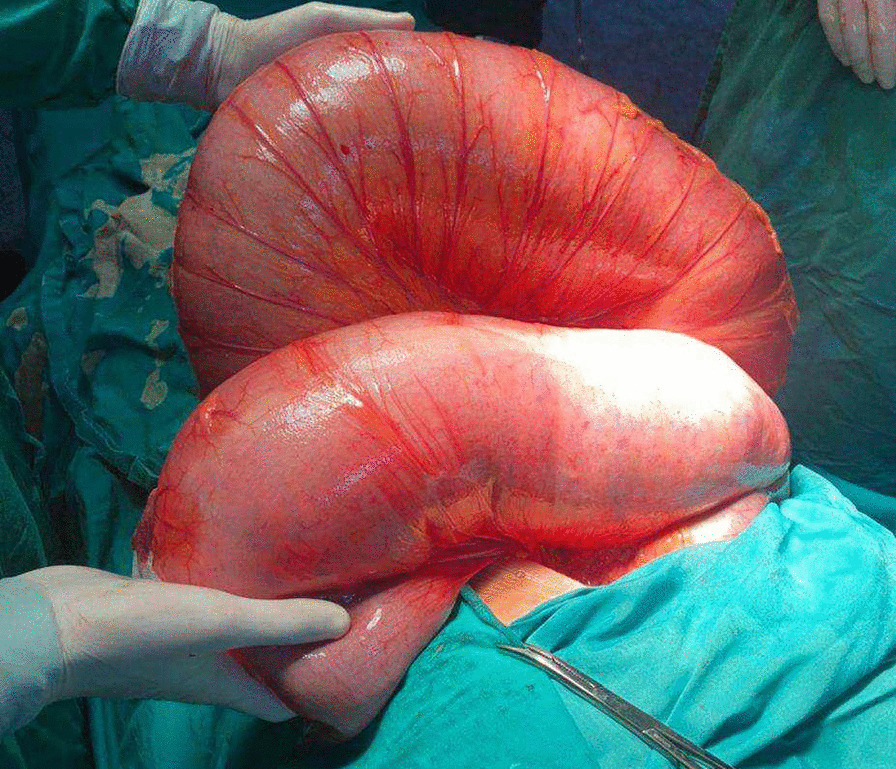
Intraoperative photo showing cecum and sigmoid colon volvulus and intensively distended colon, without any signs of perforation or ischemia

A 47-year-old male, Alawites, nonsmoker, nonalcoholic, with no history of chronic diseases, who had undergone an appendectomy through the McBurney procedure, presented with complete bowel obstruction of 5 days duration, grossly dilated abdomen, no defecation or gas passing, no specific abdominal pain, and no nausea or vomiting. On examination, vital signs were pulse 80 beats per minute, blood pressure 100/60 mmHg, and normal body temperature and respiratory rate. The abdomen was distended severely with generalized tenderness and diminished bowel sounds, and rectal examination showed an empty rectum without any intraluminal mass. Emergent laboratory data were as follows: White Blood Cell Count (WBC) 8000/mm^3^, hemoglobin 13.0 g/dL, and serum sodium and potassium levels within normal limit. Abdominal X-ray showed grossly distended large bowels with air–fluid levels (Fig. 1). A computed tomography scan (CT) showed an extremely distended large bowel. A laparotomy was performed, and the intraoperative findings were cecum and sigmoid colon volvulus and intensively distended colon, without any signs of perforation or ischemia, with parietal thinning (Fig. 2). The abdominal exploration showed the absence of all colon ligaments. A colectomy was not performed due to the refusal of the patient’s family; abdominal drains and closure were performed. The postoperative follow-up showed bowel movement after about 4 hours of operation, and the patient was discharged after 2 days.

## Case follow-up

After 8 months of the operation, the patient was admitted due to the same symptoms. An exploratory laparotomy was performed and showed sigmoid volvulus without any signs of perforation or ischemia. The sigmoidectomy (a sigmoid colectomy) was not performed due to the same reason. The postoperative course was simple, and the patient was discharged without any complications.

## Discussion

Colon volvulus (CV) is the twisting of a redundant segment of colon on its mesentery, which may lead to luminal occlusion and compromise of colonic blood supply, resulting in ischemia, gangrene, and potentially perforation [3].

The global prevalence of CV varies, with certain regions experiencing higher rates called “volvulus belt.” These regions include parts of Africa, South America, Russia, Eastern Europe, the Middle East, India, and Brazil. In this regions, colonic volvulus represents a substantial proportion of all intestinal obstructions, ranging from 13% to 42%, while in the USA and Europe, the prevalence of CV is about 10–15% of large-bowel obstruction [6]. However, for double colon volvulus, there are few reported cases to our knowledge.

Risk factors of colon volvulus are multifaceted and can be broadly categorized into lifestyle, anatomical, and medical factors. A diet low in fiber can lead to chronic constipation, which increases the risk of sigmoid volvulus. Also, sedentary lifestyle or lack of physical activity is associated with an increased risk of constipation and subsequent colon volvulus [7]. One of the possible risk factors of sigmoid colon volvulus is the length of rectum and sigmoid. The longest length was observed among African patients by Madiba *et al*. [8]. Mobile cecum—a congenital abnormality consisting of the failure of the right colon to fuse to the posterior parietal peritoneum [9]—is considered as a possible reason for recurrent right lower abdominal pain or as a misdiagnosed acute appendicitis, also a cause of cecum volvulus [10].

The main clinical presentation of CV is sudden onset of abdominal pain, which can be severe and crampy in nature. Patients often present with marked abdominal bloating or distention due to the obstruction and accumulation of gas and feces. While the obstruction progresses, nausea and vomiting may occur. If the volvulus leads to ischemia and necrosis of the bowel, signs of peritonitis, such as fever, tachycardia, and tenderness on palpation, may develop [11].

Imaging studies, particularly CT scans and abdominal X-rays, can show the “whirl sign” indicative of volvulus or the classic “coffee bean” sign in case of sigmoid volvulus [12].

The initial management in cases of sigmoid volvulus is sigmoidoscopy with application of a rectal tube for sigmoid volvulus with vital sigmoid walls [13], although surgical treatment has a higher survival rate [14] and should be considered if there are peritonitis signs or unsuccessful conservative management [15] or to prevent recurrence [16]. Colonoscopy-assisted sigmoidopexy has been shown to be an effective procedure in preventing recurrence [17], while coecopexy without resection can be done in case of vital cecum walls before the standard treatment (right hemicolectomy) in case of cecum volvulus [18].

The mortality rates are affected by age, with patients older than 75 having a high risk of mortality [19], as well as the presence of coexisting cardiopulmonary diseases and late admission [20], but there is no evidence on the association between the type of intestinal volvulus and elevated mortality risk [21].

## Conclusion

Here, we report a very rare case of double colon volvulus including the sigmoid and cecum, one of just a few cases reported in the literature. The management remains controversial and specific for every case, depending mainly on the vitality of the colonic walls and the general condition of the patient. A follow-up period is needed in conservatively treated cases of colonic volvulus to assess colon condition, performance, or late complications.

## Data Availability

Not applicable.
